# 56 The Impact of Inhalation Injury Grade on Bacterial Isolates from Diagnostic Bronchoscopy with Bronchoalveolar Lavage

**DOI:** 10.1093/jbcr/irad045.030

**Published:** 2023-05-15

**Authors:** Taylor Coston, Barclay Stewart, Devin Gaskins, Saman Arbabi, T Eoin West, John McClellan, Austin Bailey

**Affiliations:** University of Washington, Seattle, Washington; University of Washington, Seattle, Washington; University of Washington School of Medicine, Seattle, Washington; University of Washington and Harborview Medical Center, Seattle, Washington; University of Washington, Seattle, Washington; Harborview Medical Center, Tacoma, Washington; Harborview Medical Center, Rochester, New York; University of Washington, Seattle, Washington; University of Washington, Seattle, Washington; University of Washington School of Medicine, Seattle, Washington; University of Washington and Harborview Medical Center, Seattle, Washington; University of Washington, Seattle, Washington; Harborview Medical Center, Tacoma, Washington; Harborview Medical Center, Rochester, New York; University of Washington, Seattle, Washington; University of Washington, Seattle, Washington; University of Washington School of Medicine, Seattle, Washington; University of Washington and Harborview Medical Center, Seattle, Washington; University of Washington, Seattle, Washington; Harborview Medical Center, Tacoma, Washington; Harborview Medical Center, Rochester, New York; University of Washington, Seattle, Washington; University of Washington, Seattle, Washington; University of Washington School of Medicine, Seattle, Washington; University of Washington and Harborview Medical Center, Seattle, Washington; University of Washington, Seattle, Washington; Harborview Medical Center, Tacoma, Washington; Harborview Medical Center, Rochester, New York; University of Washington, Seattle, Washington; University of Washington, Seattle, Washington; University of Washington School of Medicine, Seattle, Washington; University of Washington and Harborview Medical Center, Seattle, Washington; University of Washington, Seattle, Washington; Harborview Medical Center, Tacoma, Washington; Harborview Medical Center, Rochester, New York; University of Washington, Seattle, Washington; University of Washington, Seattle, Washington; University of Washington School of Medicine, Seattle, Washington; University of Washington and Harborview Medical Center, Seattle, Washington; University of Washington, Seattle, Washington; Harborview Medical Center, Tacoma, Washington; Harborview Medical Center, Rochester, New York

## Abstract

**Introduction:**

Inhalation injury is associated with increased incidence of pneumonia. Bacteria are commonly isolated from bronchoalveolar lavage (BAL) within 12-48 hours of injury with unclear clinical significance. We aimed to determine if grade of inhalation injury is associated with different bacterial species isolated from initial BAL, and if higher inhalation injury grade is associated with increased risk of pneumonia.

**Methods:**

Abbreviated injury scores (AIS) and clinical microbiology isolates from diagnostic bronchoscopy with BAL performed within 48 hours of injury among adults with inhalation injuries from 2009-2022 were extracted from medical records at an ABA-verified burn center. CDC PNU1 criteria was used to identify pneumonia >48 hours after admission. Modified Poisson regression with robust standard errors was used to assess the association of inhalation injury grade and subsequent pneumonia.

**Results:**

Two hundred forty-six patients with inhalation injury who underwent diagnostic bronchoscopy for airway inspection and surveillance BAL within 48 hours of injury were included in this analysis. Fourteen (5.7%) were grade 0, 106 (43.1%) grade 1, 75 (30.5%) grade 2, 51 (20.7%) grade 3-4. The most common bacterial isolate was Streptococcus spp irrespective of AIS grade. Higher AIS grade was associated with increased incidence of pneumonia (IRR 1.3; 95% CI 1.09-1.61; p = 0.01) after adjustment for age, sex, and total body surface area of burn involvement.

**Conclusions:**

Bacterial species isolated did not differ by inhalation injury grade. Higher inhalation injury grade was associated with increased risk of pneumonia.

**Applicability of Research to Practice:**

Providers should be vigilant that patients with higher grade of inhalation injury are at increased risk of pneumonia. Further research is urgently needed to assess the clinical significance of organisms isolated on initial BAL and their role – if any – on the development of pneumonia.

